# Hepatoprotective, anti-inflammatory, and wound healing effects of spirulina in rats

**DOI:** 10.5455/javar.2025.l897

**Published:** 2025-03-28

**Authors:** Md. Imran Hossain, Sabbya Sachi, Purba Islam, Popy Khatun, Kazi Rafiq, Md. Zahorul Islam, Atsushi Miyamoto

**Affiliations:** 1 Department of Pharmacology, Faculty of Veterinary Science, Bangladesh Agricultural University, Mymensingh, Bangladesh; 2 Department of Veterinary Pharmacology, Joint Faculty of Veterinary Medicine, Kagoshima University, Korimoto, Japan

**Keywords:** ALT, AST, hepatoprotection, inflammation, spirulina, wound

## Abstract

**Objective:**

This study aims to investigate the hepatoprotective, anti-inflammatory, and wound-healing potentials of spirulina utilizing a rat model.

**Materials and Methods:**

Carbon tetrachloride (CCl_4_) was used to induce hepatotoxicity, while carrageenan was employed to induce hind paw inflammation. The wound healing capability was assessed by making a 6 mm round wound with a biopsy punch on the dorsal interscapular area of each rat. The animals were fed a diet mixed with spirulina at dosages of 250 and 500 mg.kg^−1^bwt. Paw thickness measurements were taken at 1, 3, and 6 h after carrageenan injection.

**Results:**

Intraperitoneal injection of CCl_4_ leads to hepatotoxicity, as evidenced by significantly elevated serum levels of alanine aminotransferase and aspartate aminotransferase. Treatment with spirulina markedly reduced these biochemical markers. CCl_4_-induced hepatic cellular necrosis, central vein congestion, and steatosis were notably improved following spirulina administration. Additionally, spirulina treatment diminished paw edema and shortened wound closure time in a dose-dependent manner. Histopathological analysis of the inflamed paw revealed massive infiltration of inflammatory cells and thickening of the epidermis, both of which showed significant improvement with spirulina treatment. The anti-inflammatory and wound-healing effects of spirulina were comparable to those of indomethacin, an established anti-inflammatory drug.

**Conclusion:**

Our findings demonstrate that *Spirulina platensis* possesses beneficial properties in counteracting hepatotoxicity and inflammation. Additionally, it exhibited significant wound-healing effects in rat models. These results reinforce the potential health advantages of spirulina as an effective functional food.

## Introduction

Spirulina is a blue-green alga that consists of approximately 30% carbohydrates, 60%–70% protein, 8% fat, essential amino acids, 3% vitamins, minerals, 3% dietary fibers, and various phytochemicals [1]. This alga has been utilized as a key ingredient in numerous biologically significant food products. Research has highlighted its antioxidant, analgesic, immune-boosting, and antibiotic properties. Additionally, Spirulina has shown beneficial effects in addressing conditions such as diabetes, obesity, hyperlipidemia, malnutrition, heavy metal toxicity, and anemia [2–4].

The liver plays a crucial role in various essential functions, including metabolism, the secretion and storage of substances, and the detoxification and elimination of harmful compounds. It is frequently exposed to hazardous chemicals and drugs. Carbon tetrachloride (CCl_4_) is a particularly toxic agent that specifically causes liver damage, leading to fatty degeneration, necrosis of liver cells, and an increased risk of liver cancer. The hepatotoxic effects, marked by the destruction of liver cells, result in elevated serum levels of aspartate aminotransferase (AST) and alanine aminotransferase (ALT) [5]. Exposure to these toxic substances generates free radicals, which contribute to hepatocellular damage [6]. In contrast, spirulina is abundant in antioxidative compounds, such as polyphenols and phycocyanin, that can neutralize the free radicals produced by hepatotoxic agents. Research demonstrates that spirulina possesses hepatoprotective properties, helping to reduce liver lipid profiles and lipid peroxidation [5].

Inflammation serves as a defense mechanism for the host against physical damage, ultraviolet radiation, microbial invasion, and immunological responses. It is typically characterized by redness, swelling, heat, and pain and can lead to exudation and impaired function. NSAIDs and corticosteroids are commonly used to mitigate inflammation; however, prolonged use of these medications is associated with adverse effects on the gut, heart, and kidneys [2,7,8]. As a result, researchers are currently exploring the effectiveness of various compounds derived from natural products. Additionally, the growing demand for health-conscious foods with nutritional benefits and disease-prevention properties has increased interest in this area. Inflammation can induce oxidative stress and diminish the antioxidant capacity of cells, suggesting that antioxidants may serve as a viable alternative for long-term anti-inflammatory therapy. Spirulina, in particular, contains several key antioxidant compounds, including carotenoids, phycocyanin, chlorophyll, phenolics, superoxide dismutase enzyme, and vitamins C and E, which help scavenge free radicals in various ways [2]. Notably, phycocyanin has demonstrated potential efficacy against drug-induced inflammation, as observed in a rat model of colitis [9]. Furthermore, spirulina has shown inflammation-inhibitory properties in human cultured intestinal epithelial cells, as well as in models of dextran sulfate-induced colitis and methotrexate-induced neurotoxicity in mice [9,10]. Antioxidants are also believed to enhance wound healing, with numerous plant-based compounds exhibiting therapeutic effects as wound-healing agents [11].

Spirulina is rich in various flavonoids, triterpenoids, and phytochemicals that are incorporated into different wound-healing dressing preparations. These components interact synergistically to promote wound healing [12]. Research has shown that spirulina enhances the wound-healing process of dermal fibroblast cells and also in mice infected with *Candida albicans* [13,14]. Today, spirulina extracts are being utilized in the cosmetic industry [15] and for addressing dermatological issues [12]. Given the extensive biological properties of spirulina, this study aims to evaluate its hepatoprotective and anti-inflammatory effects as well as wound-closing potential in rats.

## Materials and Methods

### Ethical approval

The Animal Welfare and Ethical Committee of Bangladesh Agricultural University approved [AWEEC/BAU/2022(16)] all of the experiments used in this study.

### Collection of rats

Thirty-six healthy male Wistar rats (*Rattus rattus*), weighing approximately 180 ± 5 gm and around 1 month old, were procured from the International Centre for diarrhoeal diseases research Bangladesh in Dhaka. After 1 week of acclimatization, the animals were placed in separate plastic cages according to their groups. The cages were kept in a well-ventilated room at a temperature of 28 ± 2°C, with an average humidity of 70%–80% and a natural daylight cycle. The rats had unrestricted access to a daily diet and clean drinking water *ad libitum*. They were given hand-made pellet feed, and their average feed consumption was measured previously. The composition of the hand-made pellet feed is presented in Table 1.

### Collection of spirulina and chemicals

Carrageenan and CCl_4_ were purchased from Wako Pure Chemical Corporation (Osaka, Japan) and Sigma-Aldrich Co. (St. Louis, USA), respectively. Indomethacin, sterile normal saline, and spirulina were obtained from Opsonin Pharma Limited and Radiant Pharmaceuticals Limited (Bangladesh).

### Hepatotoxicity study

Twelve rats were randomly assigned to four groups (*n* = 3): (a) Control, (b) CCl_4_ only, (c) CCl_4_ with spirulina (250 mg.kg^-1^bwt), and d) CCl_4_ with spirulina (500 mg.kg^-1^bwt). Spirulina was incorporated into the previously calculated amount of hand-made pellets for each group. All groups, except the control group, received an intraperitoneal injection of CCl_4_ (2 ml/kg in olive oil at a 1:1 ratio) to cause liver damage following 2 weeks of spirulina therapy. After a 24 h period, ketamine hydrochloride (60 mg.kg^−1^bwt) was injected (*i.p.*) to induce anesthesia. The anesthesia was maintained by diethyl ether. Blood samples were collected directly from the heart, and serum was obtained by centrifuging the clotted blood at 4000 rpm for 5 min. The resulting serum was then used for biochemical analyses, specifically ALT and AST levels. Subsequently, the experimental animals were sacrificed to obtain liver specimens. The tissues were preserved in neutral buffered formalin (10%). After that, they were dehydrated in progressively higher concentrations of alcohol, cleaned with xylene, embedded in paraffin wax, cut into slices that were 5 µm thick, and stained with hematoxylin-eosin for microscopic analysis.

**Table 1.** Composition of hand-made pellet feed.

Sl No.	Ingredients	Weight gm/kg
1	Wheat flour	690
2	Lentils	200
3	Soy flour	50
4	Fish meal	40
5	Soybean oil	12
6	Common Salt	4
7	Vitamin and mineral premix	4

### Anti-inflammatory study

Twelve rats were randomly assigned to four groups (n=3). Spirulina and indomethacin (an anti-inflammatory drug) were mixed into the daily basal diet. The groups were as follows: (a) Control, (b) Indomethacin (10 mg.kg^-1^bwt), (c) Spirulina (250 mg.kg^-1^bwt), and (d) Spirulina (500 mg.kg^-1^bwt). The treatments lasted for 2 weeks. On day fifteen, acute paw inflammation was triggered by a sub-plantar injection of freshly prepared carrageenan (100 μl, 1% in normal saline) into the left hind paws of the rats. The resulting inflammation was identified by local swelling, and the volume of edema in each paw was measured using a slide caliper at 1, 3, and 6 h after inflammation induction. The percentage of edema was calculated using the following equation:

Edema %= (Vd−Vc)/Vc ×100%

Vd is the mean volume of the paw at different times; Vc is the mean volume of the paw before carrageenan injection.

### Histological analysis

After 6 h of carrageenan injection, all the rats were euthanized to collect paw tissues. The tissues were carefully cleaned and preserved in 10% buffered formalin. The water from the fixed tissues was gradually removed using increasing concentrations of alcohol and then cleared in xylene. Subsequently, the tissues were embedded in paraffin wax. The resulting tissue blocks were sliced into 5 µm-thick sections. Microscopic observation was conducted following hematoxylin and eosin staining of the slides.

### Wound healing study

Like the anti-inflammatory study, twelve rats were randomly assigned into four groups. The animals were anesthetized using ketamine hydrochloride at a dose of 2 mg/kg body weight. The dorsal interscapular region of each rat was shaved and treated with an antiseptic solution. A circular wound measuring 6 mm in diameter was then created using a disposable biopsy punch (Kai Medical, Gifu, Japan). The wounds were left open to heal completely. Spirulina and indomethacin, an anti-inflammatory drug, were uniformly mixed into the daily feed as per the group’s requirements. The average daily feed intake for each group was calculated in advance. The required medication dose was mixed with two-thirds of the daily feed for each group. The basal diet was provided *ad libitum* after the treated feed was consumed. The wound areas were measured every third day until the ninth day post-wounding using a permanent marker and transparent paper. The rates of wound healing were calculated using this formula:

(Area of the original wound – Area of the remaining wound)/Area of the original wound ×100.

Statistical analysis

The mean ± standard error of the mean was calculated. Inter-group differences were assessed using the ANOVA test, followed by the post hoc least significant difference (LSD) test. A significance level of *p* < 0.05 was considered for all analyses. Since histological parameters were considered nonparametric, no statistical tests were conducted for these measurements.

## Results

### Effects of spirulina CCl4-induced hepatotoxicity

**Figure 1.**
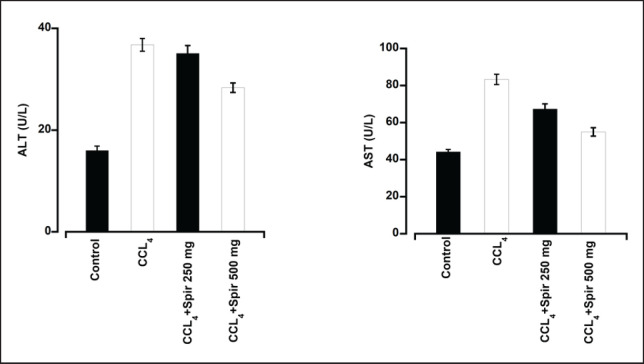
The preventative effects of spirulina (250 and 500 mg.kg^−1^bwt) on changes in serum ALT and AST levels. In rats, the intraperitoneal administration of CCl_4_ significantly increased blood ALT and AST levels, which were subsequently decreased in a dose-dependent manner through spirulina therapy. Each data point represents the mean ± SE mean from three mice.

The activities of AST and ALT were significantly elevated in the group injected with CCl_4_ (36.75 U/l and 83.34 U/l) compared to the untreated group (16 U/l and 44.28 U/l), indicating that the intraperitoneal injection of CCl_4_ caused liver damage (Fig. 1). Notably, AST and ALT levels were substantially reduced in the groups receiving spirulina at doses of 250 mg.kg^−1^ bwt (35.09 U/l and 67.41 U/l) and 500 mg.kg^-1^bwt (28.34 U/l and 55 U/l) in comparison to the CCl_4_ group (Fig. 1).

### Liver histopathology

**Figure 2.**
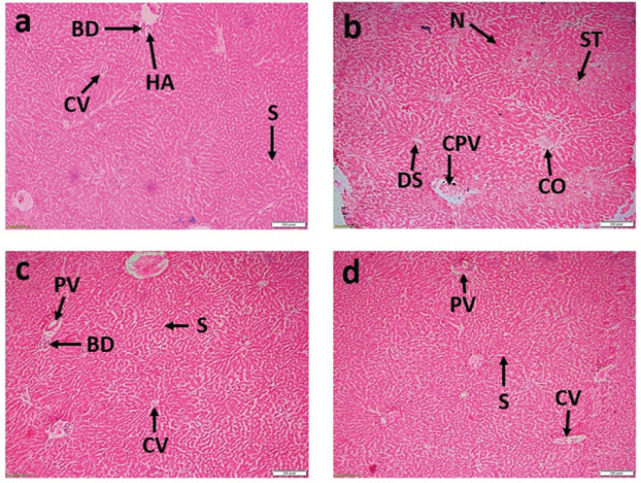
Liver tissue was stained with hematoxylin-eosin dye at a magnification of 10X. The indicated points denote: Central vein (CV), congested central vein (CO), portal vein (PV), sinusoids (S), bile duct (BD), hepatic artery (HA), congested portal vein (CPV), dilated sinusoid (DS), necrosis (N), and steatosis (ST). Liver histology observations include: (a) normal hepatocytes and central vein; (b) hepatic damage characterized by fatty degeneration, congestion, necrosis, connective tissue proliferation, and inflammatory cells observed in the CCl_4_ treatment group; (c) rats treated with CCl_4_ and spirulina at a dose of 250 mg.kg^−1^bwt exhibited regenerating hepatocytes with reduced congestion; (d) rats treated with CCl_4_ and spirulina at a dosage of 500 mg.kg^1^bwt demonstrated nearly normal liver tissue architecture.

The tissue of the control liver showed normal histological structure (Fig. 2a). In contrast, the CCl_4_ group demonstrated centrilobular necrosis, extensive hepatocyte degeneration and steatosis, vacuolation and ballooning of hepatocytes, central venous congestion, sinusoidal dilation, and significant infiltration of inflammatory cells (Fig. 2b). Interestingly, spirulina treatment dose-dependently reduced the hepatic damage caused by CCl_4_ (Fig. 2c, d).

### Effects of spirulina on carrageenan-triggered paw edema in rat

**Figure 3.**
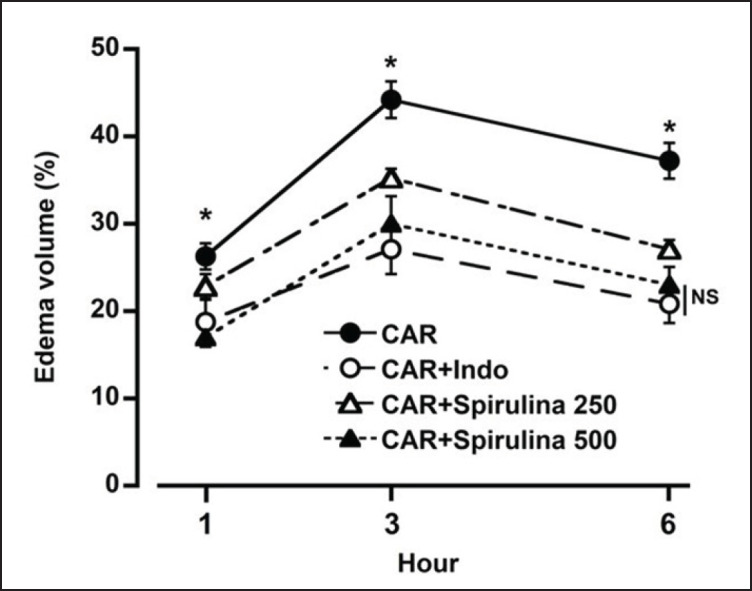
The impact of spirulina platensis on carrageenan-induced paw edema in rats. Treatment with spirulina resulted in a significant reduction in paw edema volume in a concentration-dependent manner. Notably, there was no significant difference in efficacy between indomethacin and spirulina at a dosage of 500 mg.kg^-1^bwt. Each data point represents the Mean ± SEM from three mice, with a significance level of *p < 0.05.

**Figure 4.**
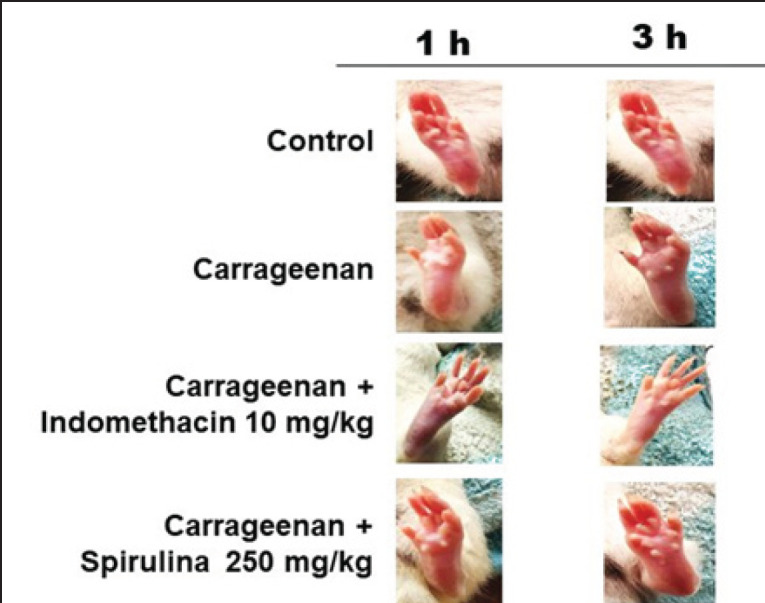
Representative images illustrating the anti-inflammatory effects of spirulina on carrageenan-induced edema in the hind paw of rats.

The injection of carrageenan resulted in a significant increase in paw volume. Treatment with spirulina demonstrated a concentration-dependent reduction in paw edema volume (Fig. 3). Paw edema was highest 3 h after injection, with approximately 45% swelling in the carrageenan-injected group, 35% in the carrageenan + spirulina 250 mg.kg^-1^bwt group, 30% in the carrageenan + spirulina 500 mg.kg^-1^bwt group, and 27% in the carrageenan + indomethacin group. At the 500 mg.kg^-1^bwt dosage, the anti-inflammatory effects of spirulina were nearly indistinguishable from those of indomethacin (Fig. 3). Figure 4 presents representative photographs of the paw edema.

**Figure 5.**
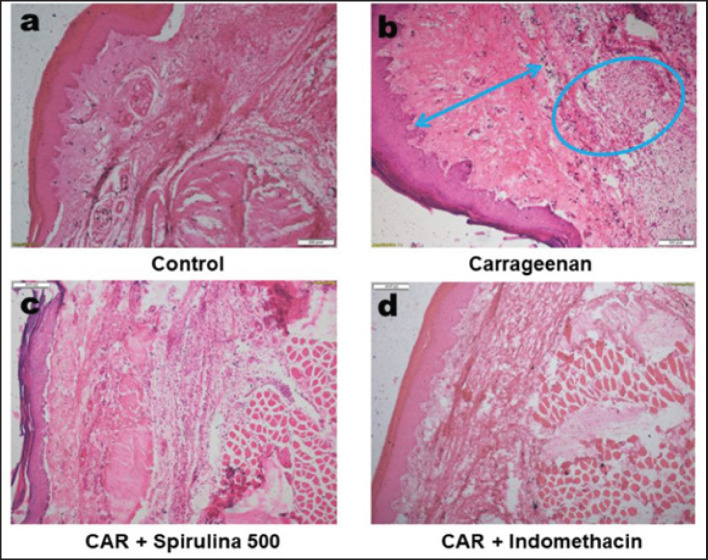
The effect of Spirulina platensis on the changes in tissue architecture of rat paws inflamed with carrageenan was examined. Tissue sections were observed after being stained with hematoxylin-eosin at 10X magnification. The control group exhibited normal tissue architecture of the rat paw (a). In the group injected with carrageenan, the arrow indicates increased dermal thickness, while the circular mark denotes the infiltration of inflammatory cells (b). In contrast, the groups treated with carrageenan plus Spirulina at a dose of 500 mg.kg^−1^bwt (c) and carrageenan plus indomethacin at 10 mg.kg^−1^bwt (d) showed a marked reduction in inflammatory cell infiltration.

**Figure 6.**
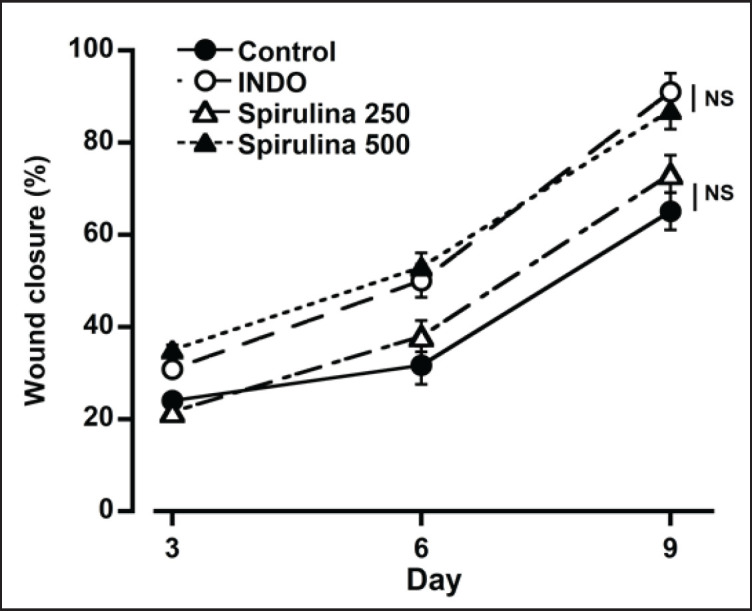
The effect of spirulina treatment (250 and 500 mg.kg^−1^bwt) on wound closure time. The results showed a significant reduction in wound area, with the effects observed to be concentration-dependent. Notably, the wound closing time associated with spirulina at a dosage of 500 mg.kg^-1^bwt was comparable to that of the commercial drug indomethacin. Each data point represents the Mean ± SEM from three mice. *NS* = not significant.

**Figure 7.**
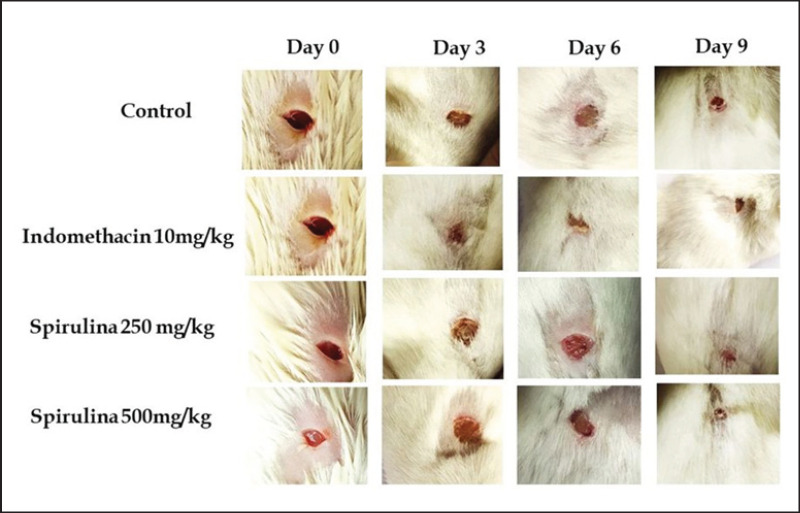
Photographic representations depict the excised wound area of rats that received treatment with spirulina at dosages of 250 and 500 mg.kg^−1^bwt and indomethacin at a dosage of 10 mg.kg^−1^bwt across various sampling days.

### Histological analyses of rat paw tissue

The paw tissue in the control group showed a normal cellular structure (Fig. 5a). In contrast, the carrageenan-treated group exhibited a marked infiltration of inflammatory cells, along with edema and dermal thickening, leading to distortion of the tissue architecture (Fig. 5b). Treatment with spirulina (500 mg.kg^-1^bwt) and indomethacin notably reduced both edema and the infiltration of inflammatory cells in the paw tissues of the rats (Fig. 5c, d).

### Effects of spirulina on wound healing

Spirulina treatment dose-dependent decreased the surface of the wound area. By day 9, the wounds in both the indomethacin (10 mg.kg^-1^bwt) and spirulina (500 mg/kg body weight) groups had almost fully healed (Fig. 6). No significant difference was observed between these two groups. On day 12, a visual assessment confirmed that all treatment groups had fully healed wounds, except the control group. Figure 7 presents representative photographs from the wound healing study.

## Discussion

Spirulina is recognized as a superfood that possesses phytochemicals exhibiting a range of pharmacological effects. Our research on rat models has revealed significant hepatoprotective, anti-inflammatory, and wound-healing properties associated with this substance.

The most well-characterized model for assessing xenobiotic-induced hepatotoxicity is CCl_4_-induced liver damage, which is commonly used to evaluate the hepatoprotective effects of new medications. The trichloromethyl radicals produced by CCl_4_ contribute to liver injury by oxidizing membrane lipids, activating hepatic Kupffer cells, and generating additional reactive oxygen species and bioactive molecules [16]. Increased levels of AST and ALT in the serum are indicators of hepatotoxicity, and significant elevations in these enzymes were observed in rats exposed to CCl_4_ [17]. In this study, exposure to CCl_4_ led to a marked rise in serum AST and ALT concentrations, signaling hepatic damage. However, in comparison to the CCl_4_-treated group, rats receiving spirulina at doses of 250 mg.kg^-1^bwt and 500 mg.kg^-1^bwt showed significantly lower levels of ALT and AST. This protective effect may be attributed to phenolic compounds in spirulina. Histological analysis of the liver tissue from the CCl_4_-treated group revealed widespread hepatocellular necrosis, fatty degeneration, extensive ballooning, and lipid degeneration. In contrast, rats receiving 500 mg.kg^-1^bwt of spirulina demonstrated liver cells that largely retained their normal architecture. These findings align with those of Osman et al. [18], who reported a pronounced hepatoprotective effect of phycocyanin, a major spirulina component, at 100, 150, and 200 mg.kg^-1^bwt doses in rats.

Carrageenan-induced hind paw edema is commonly used as a primary screening test to evaluate the anti-inflammatory effects of both natural and synthetic drugs. The study evaluated spirulina’s anti-inflammatory attributes using a common carrageenan-injected paw edema model. The carrageenan-injected paw edema was dose-dependently inhibited by the treatment of spirulina (Fig. 3). This result confirms that phytochemicals in spirulina could demonstrate anti-inflammatory properties. It has been reported that C-phycocyanin, an essential component of spirulina, showed anti-inflammatory properties by inhibiting proteinase, lipoxygenase, albumin denaturation, and hypotonicity-induced hemolysis in arachidonic acid-induced ear inflammation [19].

In this study, the histological assessment of the animal treated with carrageenan showed severe inflammation with more significant numbers of inflammatory cells (Fig. 5b). Similar research found that microscopic evaluation of the paw tissue of carrageenan-injected rats revealed significant inflammation, infiltrated inflammatory cells, and interstitial and intermuscular edema [20]. In this study, 500 mg.kg^-1^bwt of spirulina effectively reduced inflammations in rat paw tissues (Fig. 5d), a finding consistent with previous research, which revealed that spirulina supplementation significantly reduced the number of inflammatory cells in rats with carrageenan-induced paw edema [21].

Numerous plant-based compounds, including essential oils, saponins, tannins, terpenoids, flavonoids, and phenolic compounds, are utilized as natural agents for wound treatment. Phycocyanin, the most prominent pigment found in spirulina, offers a variety of medical benefits, encompassing anti-inflammatory, antioxidant, and antibacterial properties [14,22]. The application of spirulina extract has been shown to accelerate wound healing by promoting fibroblast proliferation and migration, enhancing the rate of wound closure, and reducing the growth of *Staphylococcus aureus* in wounds [23]. The wound contraction and healing effects associated with spirulina extract can largely be attributed to its ability to stimulate epithelial cell proliferation and promote angiogenesis, both of which are critical for effective wound healing. Moreover, spirulina extract appears to aid in the regeneration of the epithelium through its capacity to foster angiogenesis and facilitate collagen synthesis and deposition [24,25]. In our study, both the indomethacin 10 mg.kg^-1^bwt and spirulina 500 mg.kg^-1^bwt groups showed rapid wound healing, with no significant statistical differences between them. It is important to note that around 30%–60% of individuals treated with standard doses of indomethacin experienced side effects, and approximately 10%–20% discontinued the treatment [26]. In contrast, spirulina is recognized as a functional food with no reported adverse effects on human health. Given these attributes, it is plausible that the phenolic compounds in spirulina work synergistically to exert a potent dermal wound healing effect.

## Conclusion

The study demonstrated that spirulina possesses notable hepatoprotective, anti-inflammatory, and wound-healing properties in rats. Our research validates the positive effects of spirulina on health as a functional food. However, further researches are required to identify the specific active molecules in spirulina that have these biological effects and to understand the mechanisms of action.

## List of abbreviations

° C, degree celsius; µm, micrometer; ALT, Alanine transaminase; AST, Aspartate transaminase; bwt, Body weight; CCl_4_, Carbon tetrachloride; gm, grams; h, hour; icddr,b, International centre for diarrhoeal diseases research Bangladesh; kg, kilograms; mg, milligrams; mg.kg^-1^bwt, milligrams per kg body weight; min, minutes; ml, milliliters; mm, millimeter; NSAIDs, non-steroidal anti-inflammatory drugs; rpm, revolutions per minute; μl, microliter.
